# Physical activity, sedentary time and sleep and associations with mood states, shift work disorder and absenteeism among nurses: an analysis of the cross-sectional Champlain Nurses’ Study

**DOI:** 10.7717/peerj.8464

**Published:** 2020-03-02

**Authors:** Sonia Hajo, Jennifer L. Reed, Harleen Hans, Heather E. Tulloch, Robert D. Reid, Stephanie A. Prince

**Affiliations:** 1 Division of Cardiac Prevention and Rehabilitation, University of Ottawa Heart Institute, Ottawa, Ontario, Canada; 2 School of Human Kinetics, Faculty of Health Sciences, University of Ottawa, Ottawa, Ontario, Canada; 3 Exercise Physiology and Cardiovascular Health Lab, University of Ottawa Heart Institute, Ottawa, Ontario, Canada; 4 Faculty of Medicine, University of Ottawa, Ottawa, Ontario, Canada

**Keywords:** Physical activity, Sedentary behaviour, Sleep, Nurses, Absenteeism, Mental health

## Abstract

**Background:**

Research has suggested ideal combinations of sleep, physical activity (PA) and sedentary time (ST) (i.e., optimal sleep/high PA/low ST) are associated with better overall health. Previous research has shown nurses spend more than half their day sedentary, do not generally meet PA guidelines and have difficulty obtaining adequate sleep. There has been no known work to examine how combinations of sleep, PA and ST relate to the work performance and mental health of nurses. Our objective was to assess the associations of sleep, PA and ST with absenteeism, mood states and shift work disorder (SWD) in a sample of Canadian nurses.

**Methods:**

A total of 342 nurses from the Champlain Nurses’ Study (mean age ± SD = 43 ± 12 years, 94% women) wore an ActiGraph GT3X accelerometer for ≥ 4 days for ≥ 10 h/day to derive time spent in moderate-to-vigorous intensity physical activity (MVPA) and ST and reported sleep time for ≥ 4 days using daily activity logs. Behavioural patterns were categorized into four groups for comparison based on opposing combinations of sleep, MVPA and ST (e.g., optimal sleep/high MVPA/low ST vs. non-optimal sleep/low MVPA/high ST). Self-reported absenteeism, mood states and SWD as measured by the Profile of Mood States (POMS) and Shift Work Disorder Screening questionnaires, respectively, were compared across combinations of high versus low MVPA and ST, and optimal vs. non-optimal sleep.

**Results:**

Nurses spent an average of 444 ± 11 min/day sedentary, 14 ± 15 min/day in bouts ≥ 10 minutes of MVPA (23% met PA guidelines) and reported an average of 8 h and 39 min ± 1 h 6 min of sleep/24-h. Significant associations between behaviour groups and the POMS score and its vigor subscale, as well as SWD were observed, however, none were observed for absenteeism. The healthiest behaviour group had a significantly lower mood disturbance compared to 2/3 unhealthy behaviours and greater vigor compared to 2/3 and 3/3 unhealthy behaviours. SWD trended toward being higher amongst the group with 2/3 unhealthy behaviours. Meeting PA guidelines was associated with significantly lower total mood disturbance versus not meeting guidelines (median [IQR] = 0.4 [4.5] vs. 1.3 [4.4], *Z* =  − 2.294, *df* = 1, *p* = 0.022), as well as lower anger, higher vigor and lower fatigue. Low ST was associated with lower POMS total mood disturbance scores versus higher ST (0.6 [4.4] vs. 1.4 [4.3], *Z* = 2.028, *df* = 1, *p* = 0.043), as well as higher vigor and lower fatigue.

**Conclusions:**

In this sample of hospital nurses, the combined effects of sleep, PA and ST are associated with total mood disturbance and SWD. Achieving the recommended levels in all three behaviours may be beneficial in decreasing total mood disturbance and minimizing the effects of SWD. Future work is needed to address the low PA and high ST levels of nurses and to better understand how these behaviours can be improved to optimize the mental health of the health workforce.

## Introduction

Nurses are the largest occupational group in Canada’s health system including 282,300 publicly employed nurse managers and registered nurses (Infometrica Limited, 2017). Although nursing is perceived to be a physically active occupation, nurses have been found to spend more than half their time being sedentary on work days and very few achieve current physical activity guidelines (at least 150 min per week of moderate-to-vigorous intensity physical activity (MVPA) in bouts of 10 min or more) (Shields & Wilkins, 2006; Ratner & Sawatzky, 2009; Tremblay et al., 2011; Reed et al., 2018; Prince et al., 2019). Nurses working rotating shifts rarely obtain optimal amounts of sleep (i.e., at least 7 h) and have greater odds of reporting fair or poor health when compared to nurses who work days only (Shields & Wilkins, 2006; Rogers, 2008; Edward, 2009). Shift work, though necessary, may present a barrier to obtaining adequate sleep, being physically active and reducing sedentary time with the potential to affect nurses’ work performance and mental health (Atkinson et al., 2008).

Shift work has been linked to stress, depression, absenteeism and dissatisfaction in the workplace among nurses (Shields & Wilkins, 2006; Ferri et al., 2016). Nurses’ absenteeism rates are considerably higher than any other occupational group in Canada (Yassi, Gilbert & Cvitkovich, 2005; Shields & Wilkins, 2006; Infometrica Limited, 2017). In 2016, 24,600 nurses were absent due to their own illness or disability on a weekly basis and 61% reported taking time off for health reasons (Shields & Wilkins, 2006; Infometrica Limited, 2017). Shift workers have also been found to be at an increased risk for insomnia, chronic fatigue, anxiety, depression as well as adverse cardiovascular and gastrointestinal effects (Atkinson et al., 2008). This includes shift work disorder, a sleep disorder characterized by excessive daytime sleepiness and/or insomnia associated with shift work (Barger et al., 2012). The mechanism underlying shift work’s association with poor health may be related to changes in desynchronized circadian rhythms, sleep disturbances and psychological stress (Loprinzi, 2015). Tolerance to shift work may be affected by personality, neuroticism, rigidity of sleeping habits, commitment to shift work (how people schedule their lives) and physical fitness (Knauth & Härmä, 1992).

Sedentary time describes time spent in activities awake where the energy expenditure is ≤ 1.5 metabolic equivalents such as sitting (Tremblay et al., 2017). Greater amounts of sedentary time have been associated with lower quality and efficiency of sleep, and various health outcomes (e.g., poor metabolic profile) which are independent of physical activity levels (Loprinzi, 2015; Prince et al., 2016; Biswas et al., 2015). Currently, no Canadian guidelines for sedentary time among adults exist, but evidence has shown greater risk of cardiometabolic disease and mortality when sedentary time exceeds 8 h a day (Biswas et al., 2015; Ekelund et al., 2016; Prince et al., 2016). One study revealed nurses are approaching this value; they spend 7.5 waking hours/day being sedentary (Prince et al., 2016).

Workplace interventions, albeit few, typically focus on increasing employee physical activity levels, but fail to acknowledge the significance of addressing other health-enhancing physical behaviours (Admi et al., 2008). It is important to consider our cumulative movement behaviours and their relationship with health outcomes rather than each behaviour separately (Giacobbi, Hausenblas & Frye, 2005; Admi et al., 2008; Chaput et al., 2014; Chastin et al., 2015). Research has suggested ideal combinations of sleep, MVPA and sedentary time (i.e., optimal sleep/high MVPA, and high MVPA/low sedentary time) are associated with better overall health (Reed et al., 2018). For example, it is important that adults engage in sufficient health-enhancing physical activity, limit sedentary time (especially leisure screen time) and obtain adequate amounts of sleep (Chastin et al., 2015). Individuals who exhibit the least ideal combinations (i.e., non-optimal sleep/low MVPA/high sedentary time) are known to have less desirable measures of adiposity and cardiometabolic health; however, the effects of intermediate combinations on MVPA and mood states (e.g., tension-anxiety, depression-dejection, anger-hostility, fatigue, vigor and confusion-bewilderment) are unknown (McNair, Lorr & Droppelman, 1971; Rao, Orpana & Krewski, 2016). Emerging research has shown that when individuals engage in all three healthy movement behaviours, compared with engaging in none or only one, there are significant effects on work-related factors such as presenteeism; (Giacobbi, Hausenblas & Frye, 2005). There has been no known work to examine how combinations of sleep, physical activity and sedentary time relate to the work performance and mental health of nurses. The objective of this study was to help fill this gap in the literature by determining whether combinations of sleep, MVPA and sedentary time are associated with absenteeism, mood states and shift work disorder in a sample of Canadian nurses.

## Methods

### Study design

This is a secondary analysis of data from the cross-sectional Champlain Nurses’ Study. Details about the study have been published elsewhere (Reed et al., 2018). The study was led by the University of Ottawa Heart Institute and received ethics approval from the: Ottawa Health Sciences Network Research Ethics Board; Royal Ottawa Mental Health Centre; Children’s Hospital of Eastern Ontario; Hôpital Montfort; Renfrew Victoria Hospital; St. Francis Memorial Hospital; Queensway Carleton Hospital; Pembroke Regional Hospital; The Ottawa Hospital; Winchester District Memorial Hospital; Kemptville District Hospital; and, Cornwall Community Hospital (Protocol #: 20140670-01H; Protocol #: 15/22X; Protocol #: JR-21-01-15; Protocol #: 2015008; Protocol #: 15-04; Protocol #: 2014-003; Protocol #: 20140670-01H; Protocol #: none; Protocol #: 2014-1011; Protocol #: 20140670-01H; Protocol #: none).

### Study population

The Champlain Nurses’ study collected data from 410 nurses across 14 hospitals in the Champlain Local Health Integration Network (LHIN) of Ontario, Canada. The Champlain LHIN is one of the largest providers of public health services in the Champlain region of Ontario. All participants were recruited (via information sessions, conferences, email distribution lists, posters, social media) between December 2014 and January 2016 and provided written informed consent. Our analyses included 342 nurses with complete and valid data for MVPA, sedentary time and sleep. Analyses for the respective outcomes included complete data from 293 nurses for absenteeism, 270 nurses for shift work disorder and 301 nurses for mood states.

### Measures

#### Sleep time

Nurses self-reported sleep time over 24 h using daily activity log sheets. Nurses were instructed to complete daily activity logs by indicating in writing when they went to sleep and when they woke up. Total sleep time was then calculated by taking the sum of hours between the indicated sleep time(s) and wake time(s) which includes any time spent napping. Total sleep time and number of sleep bouts were reported. The total value for sleep time was used in the analysis for this study. A daily sleep duration of 7–9 h has been identified as optimal and associated with overall good health status in adults and older adults (Hirshkowitz et al., 2015). Participants were dichotomized into having optimal sleep levels (7–9 h of sleep per 24-hours) compared to those with non-optimal sleep levels (less than 7 h or more than 9 h per 24-hours) (Hirshkowitz et al., 2015).

#### Moderate-to-vigorous intensity physical activity (MVPA)

MVPA was objectively measured using ActiGraph GT3X accelerometers (Actigraph, Pensacola, Florida). Accelerometers were worn over the right hip for a nine-day period excluding water-related activities (e.g., bathing, swimming). Hip worn accelerometry has been shown to be superior for predicting locomotion when compared to other device positions, such as the non-dominant wrist (Migueles et al., 2017). The ActiGraph GT3X has shown to be reliable and valid in reporting a range of intensities of physical activity (Migueles et al., 2017). A 15-second sampling epoch was utilized and converted to counts-per-minute (cpm). To be considered valid, participants were required to have ≥ 10 h per day and ≥ 4 days of wear-time (Colley et al., 2011). A maximum of seven days of data was used (first and last days were removed to reduce potential reactivity to wearing the devices). Non-wear time was defined as ≥ 60 min of consecutive zeros for counts, with an allowance of up to two minutes of counts between zero and 150. MVPA was defined using a validated cut-point of ≥ 2691 cpm using vector magnitude (Sasaki, John & Freedson, 2011). Weekly MVPA was calculated by multiplying the daily average by seven. Participants were characterized as meeting the Canadian Physical Activity Guidelines if they had ≥ 150 min of MVPA per week in bouts of ≥ 10 min (with an allowance of two-minutes of disruption) (Tremblay et al., 2011).

#### Sedentary time

Sedentary time was also objectively measured using the accelerometers and categorized using a validated vector magnitude cut-point of ≤ 150 cpm (Peterson et al., 2015). Although sedentary time guidelines/thresholds are not currently available in Canada or internationally, participants were dichotomized into those having high levels of sedentary time (≥ 8 h) compared to those with lower levels of sedentary time (< 8 h) given previous associations with health risk (Ekelund et al., 2016).

#### Shift work disorder

Nurses self-reported shift work disorder using the Shift Work Disorder Screening Questionnaire (Barger et al., 2012). The four-item questionnaire uses 4- and 5-point Likert scales yielding scores ranging from 4–17, with lower and higher scores indicating the absence or presence of shift work disorder, respectively (Barger et al., 2012). The questionnaire has been shown to correctly identify 76% of previously diagnosed cases of shift work disorder with a sensitivity and specificity of 0.74 and 0.82, respectively (Barger et al., 2012). The analysis of shift work disorder excluded nurses who reported working only day shifts.

#### Absenteeism

Nurses’ absenteeism was assessed using the Health and Work Performance Questionnaire (short-form) designed by the World Health Organization and has been shown to have high concordance with real life measures of workplace absenteeism (Kessler et al., 2003). The questionnaire comprises seven questions to evaluate the effect of health problems on absenteeism in the workplace (Kessler et al., 2003). It provides a full range of continuous scores for seven days or four weeks, and absolute or relative absenteeism depending on hours and days missed and expected from work (Kessler et al., 2003). It includes 10-point Likert scale questions asking respondents how often, during their working hours, they had decrements in quantity and quality of work, with 1 being the worst performance and 10 being the best performance (Kessler et al., 2003). Relative absenteeism is based on the difference between the number of hours an employee is expected to work, and the number of hours actually worked over a 4-week period (Kessler et al., 2004). We used 4-week relative absenteeism as opposed to 7-day estimates as weekly absenteeism may not reflect usual habits (e.g., missed work hours due to a doctor’s appointment) (Kessler et al., 2004).

#### Mood states

Mood states were assessed using the Profile of Mood States (POMS) questionnaire (McNair, Lorr & Droppelman, 1971). The questionnaire contains 65 items using 5-point Likert scales ranging from 0 (not at all) to 4 (extremely) (McNair, Lorr & Droppelman, 1971). For the purpose of this study continuous total mood disturbance scores were used. Total mood disturbance is calculated by adding all subscales (tension-anxiety, depression-dejection, anger-hostility, fatigue-inertia, confusion-bewilderment) and subtracting vigor-activity; scores can range from 0 to 200, where higher scores indicate a greater degree of mood disturbance (McNair, Lorr & Droppelman, 1971). The POMS questionnaire is a valid measure for assessment of mood swings over a period of time in the workplace environment and demonstrates consistency, validity and reliability for measures of mood (Morfeld et al., 2007; Wyrwich & Yu, 2011).

### Statistical analysis

**Figure 1:**
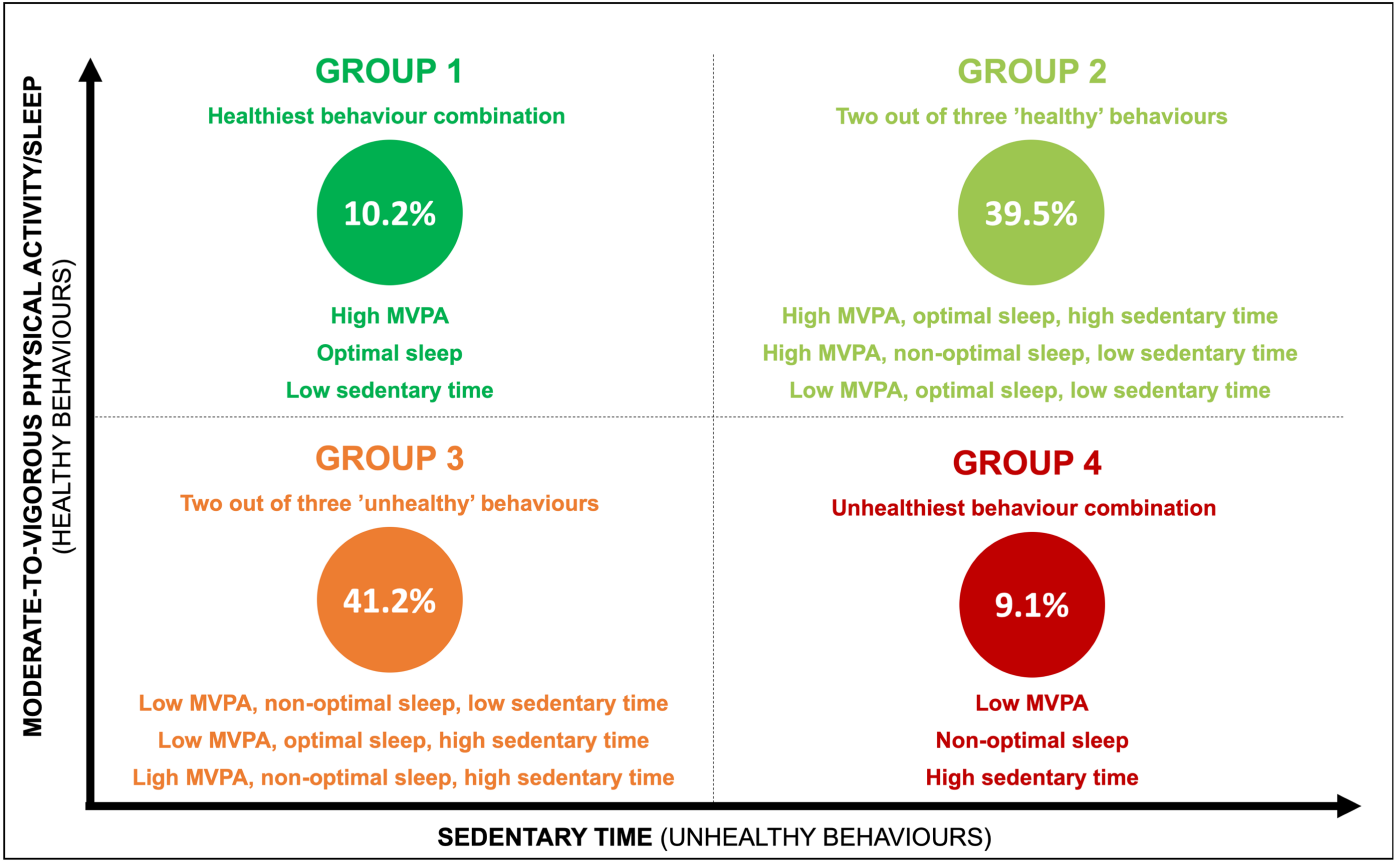
Four behavioural groups. MVPA, moderate-to-vigorous intensity physical activity.

All analyses were conducted using SPSS v24 (IBM Corp, NY, USA). Univariate analyses were used to produce descriptive results including means (standard deviations) and proportions. Between groups differences by sex and for missing/non-missing data were examined using independent t-tests and chi-square tests. Analyses were conducted using data from nurses with complete data (nurses with complete data were no different than those with incomplete data). Data were tested for normality visually using plots and Shapiro–Wilks test of normality. Behavioural patterns were categorized into four groups (see Fig. 1) to compare combinations of ‘more healthy’ and ‘less healthy’ behaviours. Kruskal–Wallis one-way analyses of variance were used to compare mean outcomes for absenteeism and POMS across all four behavioural comparison groups with multiple comparisons conducted using a Bonferroni correction. Chi-square tests were used to compare shift work disorder across the four groups using adjusted residuals for multiple comparisons with *p* < 0.006 required for significance. As none of the variables of interest were normally distributed, non-parametric tests (independent samples Mann–Whitney U tests) were used for two-way comparisons between absenteeism/POMS and MVPA, sedentary time and sleep. Chi-square tests were used for two-way comparisons between shift work disorder and all three movement behaviours.

## Results

A total of 342 nurses (83% of overall sample) from the Champlain Nurses’ Study had complete MVPA, sedentary time and sleep data; 159 had valid data and worked non-day shifts. Characteristics of this sample of nurses are shown in Table 1. Nurses’ mean age was 43 ± 12 years and 94% were women. The majority were married (68%), of White ethnicity (87%), considered overweight (56%), worked 8-hour shifts (56%), and in urban hospital locations (82%). Half worked fixed shifts (days, evenings or nights only) with most working fixed day shifts. No statistically significant differences in participant characteristics, exposures or outcomes were observed between men and women (*p* > 0.05 each). Complete versus missing data for all outcomes (shift work disorder, absenteeism, POMS) did not significantly differ by age, sex, shift length, shift type, sleep, sedentary time or MVPA. Missing data for the POMS questionnaire were more likely to come from nurses working in urban hospitals. Additionally, missing data for shift work disorder were more likely to be from nurses working 8-hour and fixed shifts.

**Table 1:** Participant characteristics (*N* = 342).

	Mean ± SD or *n* (%)
**Demographics and anthropometrics**^a^	
Age (years)	43.1 ± 11.8
Sex, female	323 (94%)
Ethnicity	
White	295 (86%)
Black	20 (6%)
Asian	14 (4%)
Other/missing	13 (4%)
Marital status	
Married or equivalent	234 (68%)
Single	81 (24%)
Separated	24 (7%)
Widowed	3 (1%)
Education	
College certificate or diploma	129 (38%)
University bachelor’s degree	168 (49%)
University certificate above bachelor	45 (13%)
Personal annual income (CAD) (*n* = 336)	
< $50,000	32 (10%)
$50,000–$74,999	111 (33%)
≥ $75,000	193 (57%)
Shift type (*n* = 340)	
Fixed (days, evenings, nights)	170 (50%)
Rotating (days and nights, days, nights and evenings, days and evenings)	170 (50%)
Smoker	26 (8%)
Menopausal (women only)	5 (1.5%)
Height (cm)	164.4 ± 6.6
Body mass (kg)	73.0 ± 15.1
BMI (kg/m^2^)	27.0 ± 5.5
Waist circumference (cm)	80.6 ± 11.8
Resting systolic blood pressure (mmHg)	114.5 ± 12.6
Resting diastolic blood pressure (mmHg)	74.0 ± 8.6
Resting heart rate (bpm)	68.6 ± 9.6
**Medical conditions (where medication has been prescribed)**^b^	
Musculoskeletal pain, injury or repair	56 (16%)
Depression	31 (9%)
Anxiety	24 (7%)
Asthma	28 (8%)
Hypertension	23 (7%)
Hypothyroidism	20 (6%)
Gastrointestinal issues (e.g., GERD, gastritis, Crohn’s)	20 (6%)
Arthritis	16 (5%)
Sleep apnea	9 (3%)
Dyslipidemia	13 (4%)
Headaches or migraines	13 (4%)
Diabetes	6 (2%)
Skin disorder (psoriasis, eczema, dermatitis)	7 (2%)
Allergies	6 (2%)
Anemia	4 (1%)
Hormonal therapy	4 (1%)

**Notes.**

BMI Body mass index GERD Gastroesophageal reflux disease SD Standard deviation CAD Canadian dollars

a Only the most frequently reported ethnicity, marital status, level of education and personal annual income are reported.

b Only medical conditions for which more than 1% of participants were taking medications are reported.

Nurses spent an average of 444 ± 11 minutes/day sedentary (70% sedentary for < 8 hours/day), 14 ± 15 minutes/day in bouts ≥ 10 min of MVPA (23% meeting MVPA guidelines) and reported an average of 8 h 39 min ± 1 h 5 min of sleep (58% were ‘optimal’ sleepers) and 1.2 ± 0.3 sleep-wake cycles per 24-hour period (Table 2). On average, nurses had 8 valid sleep log entries (range of 4-9 entries). None of the covariates were significantly associated with the exposures and outcomes. Of the four comparison groups, 10.2% of nurses were characterized as belonging to Group 1, 39.5% belonging to Group 2, 41.2% belonging to Group 3 and 9.1% belonging to Group 4. Approximately half the sample achieved at least two of three healthy behaviours (i.e., high MVPA/high sedentary time/optimal sleep, high MVPA/low sedentary time/non-optimal sleep or low MVPA/low sedentary time/optimal sleep) (Fig. 1).

**Table 2:** Nurses’ movement behaviours (*n* = 342).

	**Mean ± SD or *n* (%)**
**Self-reported sleep time**	
Total sleep time per 24-hour period	8 h 39 min ± 1 h 5 min
Average sleep-wake cycles per 24-hour period	1.2 ± 0.3
**Objectively measured sedentary time, physical activity levels, and steps**	
Light-intensity physical activity, minutes/day	409.6 ± 78.5
Moderate-intensity physical activity, minutes/day	38.2 ± 18.5
Vigorous-intensity physical activity, minutes/day	3.4 ± 5.5
MVPA, minutes/day	41.5 ± 20.6
Steps, number/day	8241 ± 2348
MVPA, minutes/week	290.8 ± 144.32
MVPA in bouts, minutes/week	97.4 ± 101.3
Meeting MVPA guidelines	90 (23.4)
Sedentary time, minutes/day	443.7 ± 111.2

**Notes.**

MVPA Moderate-to-vigorous intensity physical activity SD standard deviation

No significant difference between the four behavioural comparison groups (combinations of MVPA, sedentary time and sleep) were found for absenteeism (Table 3). Significant associations between behaviour groups and POMS total mood disturbance and vigor subscale and shift work disorder were observed. Group 1 had a significantly lower mood disturbance compared to Group 3 and greater vigor compared to Group 3 and Group 4. Pairwise comparisons for shift work disorder did not identify specific between-group differences. Meeting the MVPA guidelines was associated with significantly lower POMS scores (i.e., lower mood disturbance) (median [IQR] = 0.4 [4.5] vs. 1.3 [4.4], *Z* =  − 2.294, *df* = 1, *p* = 0.022), lower anger, higher vigor and lower fatigue compared to not meeting the guidelines (Table 4). Low sedentary time was also associated with lower POMS scores (median [IQR] = 0.6 [4.4] vs. 1.4 [4.3], *Z* = 2.028, *df* = 1, *p* = 0.043), higher vigor and lower fatigue versus higher sedentary time (Table 4). Significant and negative correlations were observed between sedentary time and sleep (*ρ* = − 0.244, *p* < 0.001), and MVPA in bouts and sedentary time (*ρ* = − 0.112, *p* = 0.038).

**Table 3:** Between group differences in POMS, relative absenteeism and shift work disorder by four combination groups of MVPA, sedentary time and sleep.

	Group 1	Group 2	Group 3	Group 4	Test statistic	*p-*value
POMS^a^^,^^c^ *n* = 301	0.3 [4.4]	0.4 [4.2]	1.9 [4.9]	1.7 [3.3]	*χ*^2^= 11.303, *df* = 3	**0.010**
Tension^a^	0.5 [0.8]	0.7 [0.8]	0.8 [0.7]	0.7 [0.7]	*χ*^2^= 3.928, *df* = 3	0.269
Anger^a^	0.3 [0.7]	0.3 [0.6]	0.4 [0.6]	0.4 [0.6]	*χ*^2^= 5.489, *df* = 3	0.139
Depression^a^	0.1 [0.5]	0.3 [0.6]	0.4 [0.8]	0.3 [0.4]	*χ*^2^= 5.267, *df* = 3	0.153
Confusion^a^	0.2 [0.8]	0.4 [0.6]	0.6 [0.6]	0.6 [0.6]	*χ*^2^= 5.111, *df* = 3	0.164
Vigor^a^^,^^d^^,^^e^	2.3 [1.5]	2.2 [1.2]	1.8 [1.3]	1.7 [1.5]	*χ*^2^= 13.891, *df* = 3	**0.003**
Fatigue^a^	1.0 [1.2]	0.8 [1.4]	1.2 [1.8]	1.2 [1.4]	*χ*^2^= 7.604, *df* = 3	0.055
Relative absenteeism^a^ *n* = 289	0.3 [24.8]	0 [24.8]	1.3 [25.0]	0 [20.9]	*χ*^2^= 2.193, *df* = 3	0.533
Shift work disorder^b^ *n* = 159	27.3%	51.5%	62.1%	31.3%	*χ*^2^= 8.170, *df* = 3	**0.043**

**Notes.**

a Kruskal–Wallis one-way analysis of variance with multiple comparisons using a Bonferroni correction, data presented as median [interquartile range].

b Chi-square test. df, degrees of freedom; POMS, Profile of Mood States; MVPA, moderate-to-vigorous intensity physical activity.

c Group 3 is greater than Group 1.

d Group 1 is greater than Group 4.

e Group 1 is greater than Group 3.

**Table 4:** Between group differences in POMS, absenteeism and shift-work disorder by low versus high MVPA, sedentary time and sleep.

			Test statistic	*p*-value
	**Moderate-to-vigorous intensity physical activity**			
	**High** (≥ 150 minutes/week, in bouts of ≥ 10 min)	**Low** (< 150 minutes/week, in bouts of ≥ 10 min)		
POMS (*n* = 301)^a^	0.4 [4.5]	1.3 [4.4]	*Z* = − 2.29, *df* = 1	**0.022**
Tension^a^	0.5 [0.8]	0.7 [0.7]	*Z* = − 1.51, *df* = 1	0.132
Anger^a^	0.3 [0.6]	0.4 [0.7]	*Z* = − 1, 98, *df* = 1	**0.048**
Depression^a^	0.1 [0.6]	0.4 [0.6]	*Z* = − 1.92, *df* = 1	0.055
Confusion^a^	0.4 [0.8]	0.4 [0.6]	*Z* = − 0.90, *df* = 1	0.369
Vigor^a^	2.2 [1.3]	2.0 [1.2]	*Z* = 2.15, *df* = 1	**0.031**
Fatigue^a^	1.0 [1.3]	1.0 [1.4]	*Z* = − 2.12, *df* = 1	**0.034**
Absenteeism (*n* = 289) ^a^	0.0 (20.4)	1.3 (22.9)	*Z* = 1.29, *df* = 1	0.196
Shift work disorder (*n* = 159)^b^				
High risk	39.3%	55.0%	*χ*^2^= 2.27, *df* = 1	0.132
Low risk	60.7%	45.0%		
	**Sedentary time**			
	**High** (≥ 8 h)	**Low** (< 8 h)		
POMS (*n* = 301)^a^	1.4 [4.3]	0.6 [4.4]	*Z* = 2.028, *df* = 1	**0.043**
Tension^a^	0.7 [0.7]	0.7 [0.7]	*Z* = 0.441, *df* = 1	0.659
Anger^a^	0.4 [0.7]	0.4 [0.6]	*Z* = 1.582, *df* = 1	0.114
Depression^a^	0.3 [0.5]	0.3 [0.6]	*Z* = 0.999, *df* = 1	0.318
Confusion^a^	0.6 [0.6]	0.4 [0.6]	*Z* = 1.011, *df* = 1	0.312
Vigor^a^	1.8 [1.3]	2.2 [1.3]	*Z* = − 2.761, *df* = 1	**0.006**
Fatigue^a^	1.2 [1.6]	1.0 [1.4]	*Z* = 2.034, *df* = 1	**0.042**
Absenteeism (*n* = 289) ^a^	0.0 [20.3]	1.3 [24.8]	*Z* = 0.207, *df* = 1	0.836
Shift work disorder (*n* = 159)^b^				
High risk	51.3%	52.5%	*χ*^2^ = 0.017, *df* = 1	0.895
Low risk	48.7%	47.5%		
	**Sleep**			
	**Optimal** (7–9 h)	**Non-optimal** (< 6 and > 9 h)		
POMS (*n* = 301)^a^	1.2 [4.4]	1.1 [4.2]	*Z* = − 1.483, *df* = 1	0.138
Tension^a^	0.7 [0.7]	0.5 [0.8]	*Z* = − 0.536, *df* = 1	0.592
Anger^a^	0.3 [0.7]	0.3 [0.6]	*Z* = 0.316, *df* = 1	0.752
Depression^a^	0.4 [0.6]	0.4 [0.6]	*Z* = − 0.472, *df* = 1	0.637
Confusion^a^	0.4 [0.6]	0.4 [0.8]	*Z* = − 1.934, *df* = 1	0.053
Vigor^a^	2.2 [1.2]	1.8 [1.2]	*Z* = 1.635, *df* = 1	0.102
Fatigue^a^	1.0 [1.6]	1.2 [1.4]	*Z* = − 0.763, *df* = 1	0.445
Absenteeism (*n* = 289)^a^	1.3 [21.6]	1.3 [25.1]	*Z* = 0.965, *df* = 1	0.335
Shift work disorder (*n* = 159)^b^				
High risk	53.0%	51.3%	*χ*^2^= 0.046, *df* = 1	0.831
Low risk	47.0%	48.7%		

**Notes.**

a Independent samples Mann–Whitney U test.

b Chi-square test. Df, degrees of freedon; POMS, Profile of Mood States.

Values are medians [interquartile range] unless otherwise stated.

## Discussion

This was the first study, to our knowledge, to assess the synergistic associations of sleep, sedentary time and MVPA with absenteeism, shift work disorder and mood states among a large sample of hospital nurses. On average, sleep time was within the recommended 7 to 9 h of sleep, sedentary time was slightly below the 8-hour threshold, but MVPA was substantially lower than the recommended 150 min per week when using ≥ 10-minute bouts. We observed significant differences in total mood disturbance by health behaviour groups, however, no significant differences between groups were found for absenteeism or shift work disorder.

Group 1 (most optimal combination of health behaviours) had a significantly lower mood disturbance compared to Group 3 (2/3 unhealthy behaviours) and greater vigor compared to Group 3 and Group 4 (all unhealthy behaviours). A closer look at differences in individual behaviours found lower total mood disturbance (higher POMS scores) and affected mood subscales including lower anger, higher vigor and lower fatigue in those who met MVPA guidelines compared to those who did not meet guidelines. We also observed lower mood disturbance in those with less than 8 h of sedentary time compared to those with greater than 8 h of sedentary time including higher vigor and lower fatigue. These findings are consistent with a previous study which found that within the composition of movement behaviours, physical activity has the strongest positive association with better health outcomes (Chastin et al., 2015).

Contrary to our hypothesis, we did not find a significant difference in nurses’ levels of absenteeism and shift work disorder across the four groups. Given that these nurses volunteered to participate in this study, it is possible that they represent a biased sample of younger, healthier individuals with a reduced likelihood of absenteeism and shift work disorder.

Given that the number of hours in a day is finite, this leads us to believe that time spent in one behaviour is co-dependent of time spent in others (Colley, Michaud & Garriguet, 2018). We found that high sedentary time was related to less sleep while lower sedentary time was related to greater MVPA. These associations demonstrate that the behaviours are not entirely independent. The effect of time spent in one behaviour may, therefore, depend on the composition of the rest of the day and may also displace time spent in another behaviour. Of this sample, 197 (57.6%) nurses obtained optimal levels of sleep and 239 (70%) nurses engaged in less than 8 h of sedentary time, showing that the majority of nurses achieved what is considered healthier levels of sleep and sedentary time (Edward, 2009; Ekelund et al., 2016). Only 23% of nurses had adequate/higher levels of MVPA, which is likely why we observed the strongest positive association with mood state.

A closer look at this sample revealed that mental health conditions such as depression (9.1%) and anxiety (7.0%) were prevalent (Reed et al., 2018). Findings from this study revealed that nurses’ MVPA levels were significantly related to measures of mood states, which can potentially affect their mental health. When nurses had higher levels of MVPA, they reported better mood state scores. It is well established that increased levels of physical activity can significantly increase positive mood states and reduce negative mood (Giacobbi, Hausenblas & Frye, 2005). The literature suggests different exercise intensities (e.g., moderate intensity/high frequency and high intensity/moderate frequency) may engender more positive mood states and mental health through a dose–response relationship (Katula, Blissmer & McAuley, 1999; Shephard, 2001). Previous research has shown that physical activity plays a role in regulating stress and psychological well-being in shift workers (medical interns) in health care (Kalmbach et al., 2018). These workers have reported poorer mood when they are less active, therefore, a reduction in physical activity may increase the risk for poorer mood outcomes (Kalmbach et al., 2018).

There is an increased interest in the association between time spent being sedentary and mental health, however, this field is in its infancy (Hamer & Smith, 2018). Findings from this study revealed that nurses’ sedentary time was significantly associated with higher POMS total mood disturbance scores. When nurses were less sedentary (< 8 hours/day), they had lower mood disturbance compared to those who were highly sedentary (≥ 8 hours/day). Emerging research has also shown a consistent association between greater sedentary time and poorer mental health, independent of changes in MVPA (Hamer, Coombs & Stamatakis, 2014; Endrighi, Steptoe & Hamer, 2016). The mechanisms underlying the effect of sedentary time on mental health are not completely understood but may be associated with enhanced pro-inflammatory responses due to acute stress and transient mood disturbances induced by greater time spent sedentary (Endrighi, Steptoe & Hamer, 2016). Past interventions targeting sedentary behaviour demonstrate that decreased sedentary time can improve mental well-being and may benefit mood, stress and sleep (Ellingson et al., 2018).

### Limitations

This study has limitations that warrant mention. Firstly, there are no guidelines for sedentary time, therefore, we applied a cut-off previously associated with poor health outcomes (Ekelund et al., 2016). This threshold may have misclassified some of the sample. Secondly, sleep was self-reported by the nurses and can, therefore, be affected by response or recall bias. Previous research has shown that self-reported sleep only moderately aligns with objective measures (Jackowska et al., 2016). Generalizability of our findings to male nurses is also limited as 94% the sample consisted of women; however, this is characteristic of Canada’s population of registered nurses (Infometrica Limited, 2017). These findings may not be generalizable to younger nurses given that the sample consisted largely of middle-aged nurses (mean age 43.1 ± 11.8 years, 98.5% pre-menopausal). This sample did, however, have a good distribution of nurses working various roles (e.g., administrative, clinical) from both urban and rural hospital locations and who worked different lengths and types of shifts (e.g., fixed, rotating, days, nights, evenings, 8-, 12- and a combination of 8- and 12-h shifts). Finally, this was a cross-sectional study; therefore, we are unable to establish a causal relationship between MVPA, sedentary time and sleep, and absenteeism, mood state and shift work disorder.

## Conclusion

Emerging research has revealed the importance of healthy movement behaviours and the relationship between ideal combinations of sleep, MVPA and sedentary time and mental-health related outcomes. There is less work looking at the combined effects of these behaviours on aspects of work performance and mental health, especially in the workplace. In our sample of Canadian nurses, combinations of these behaviours were significantly associated with mood state, however, they were not significantly associated with absenteeism or shift work disorder. Furthermore, nurses that met physical activity guidelines and had lower levels of sedentary time had significantly lower total mood disturbance (better mood state scores). Our findings underscore the importance of nurses achieving optimal levels in all three behaviours, in particular, obtaining adequate levels of MVPA and limiting sedentary time, as this may be beneficial in decreasing total mood disturbance and minimizing the effects of shift work disorder. Future work is needed to address the lower physical activity and high sedentary behaviour levels of nurses and to better understand how these behaviours can be improved to optimize the mental health of the health workforce.

## Additional Information and Declarations

### Data Availability

The following information was supplied regarding data availability: The Ottawa Health Science Network Research Ethics Board has placed restrictions on data access. For researchers who meet the criteria for access to confidential information, data are available from Dr. Jennifer Reed through the Ottawa Health Science Network Research Ethics Board.
